# The diagnosis, burden and prognosis of dementia: A record-linkage cohort study in England

**DOI:** 10.1371/journal.pone.0199026

**Published:** 2018-06-26

**Authors:** Mar Pujades-Rodriguez, Valentina Assi, Arturo Gonzalez-Izquierdo, Tim Wilkinson, Christian Schnier, Cathie Sudlow, Harry Hemingway, William N. Whiteley

**Affiliations:** 1 Leeds Institute of Health Sciences, University of Leeds, Leeds, United Kingdom; 2 Usher Institute of Population Health Sciences and Informatics, University of Edinburgh, Edinburgh, United Kingdom; 3 Institute of Health Informatics, University College London, London, United Kingdom; 4 Health Data Research UK London, London, United Kingdom; 5 Health Data Research UK Scotland, Edinburgh, United Kingdom; 6 Centre for Clinical Brain Sciences, University of Edinburgh, Edinburgh, United Kingdom; Istituto Di Ricerche Farmacologiche Mario Negri, ITALY

## Abstract

**Objectives:**

Electronic health records (EHR) might be a useful resource to study the risk factors and clinical care of people with dementia. We sought to determine the diagnostic validity of dementia captured in linked EHR.

**Methods and findings:**

A cohort of adults in linked primary care, hospital, disease registry and mortality records in England, [CALIBER (CArdiovascular disease research using LInked Bespoke studies and Electronic health Records)]. The proportion of individuals with dementia, Alzheimer’s disease, vascular and rare dementia in each data source was determined. A comparison was made of symptoms and care between people with dementia and age-, sex- and general practice-matched controls, using conditional logistic regression. The lifetime risk and prevalence of dementia and mortality rates in people with and without dementia were estimated with random-effects Poisson models. There were 47,386 people with dementia: 12,633 with Alzheimer’s disease, 9540 with vascular and 1539 with rare dementia. Seventy-four percent of cases had corroborating evidence of dementia. People with dementia were more likely to live in a deprived area (conditional OR 1.26;95%CI:1.20–1.31 most vs least deprived), have documented memory impairment (cOR = 11.97;95%CI:11.24–12.75), falls (cOR = 2.36;95%CI:2.31–2.41), depression (cOR = 2.03; 95%CI:1.98–2.09) or anxiety (cOR = 1.27; 95%CI:1.23–1.32). The lifetime risk of dementia at age 65 was 9.2% (95%CI:9.0%-9.4%), in men and 14.9% (95%CI:14.7%-15.1%) in women. The population prevalence of recorded dementia increased from 0.3% in 2000 to 0.7% in 2010. A higher mortality rate was observed in people with than without dementia (IRR = 1.56;95%CI:1.54–1.58).

**Conclusions:**

Most people with a record of dementia in linked UK EHR had some corroborating evidence for diagnosis. The estimated 10-year risk of dementia was higher than published population-based estimations. EHR are therefore a promising source of data for dementia research.

## Introduction

Dementia is a common progressive clinical syndrome that develops slowly over years. Many of those affected are disabled not only by cognitive impairment but also by common co-morbidities of ageing such as stroke, arthritis, and heart disease. The burden of dementia on patients, carers and the health system is substantial, and might increase as populations grow older.[1]

Very long follow-up is needed to study risk factors for dementia, because of the long prodrome before suspicion of diagnosis, reverse causality, and because it is likely that different factors affect disease risk at different stages over a lifetime. This means that well conducted prospective studies with complete follow-up are important, but they are rare and costly.[2,3] More efficient methods to study dementia, on a large scale and with a low dropout rate, would improve our understanding of the pathophysiology of this condition. National electronic health records (EHR) linking primary care, hospital and death records are a potentially important source of data for dementia research. They include people with the severest manifestations of dementia or disability, who might be under-represented in bespoke recruited cohorts because they are difficult to recruit or follow-up. They also capture a wide variety of important health events. However, it is uncertain whether EHRs capture dementia with sufficient accuracy and completeness.

Studies of the validity of dementia in EHRs reported positive predictive values for a dementia of up to 90% [4,5] (i.e. when a diagnosis is recorded, people usually have dementia). These studies have relied on hand searching of individual clinical charts, and therefore had modest sample sizes (<500). EHR might underestimate the proportion of people with dementia (i.e. a low sensitivity) compared with bespoke cohorts. However, the longitudinal nature of electronic medical records provides multiple opportunities for capture of a dementia diagnosis, and therefore measurement of the lifetime risk of dementia could provide a better measure of the sensitivity of EHRs for dementia diagnosis.

In this study, we sought to determine how dementia is captured in different routinely collected medical data sources; whether characteristic dementia symptoms might improve dementia ascertainment; and to determine the lifetime risk of dementia from these records.

## Methods

### Study population

We studied a cohort of people registered in the Clinical Practice Research Datalink (CPRD) general practices between 1^st^ January 1998 and 31^st^ March 2010. At study entry, eligible patients were aged 18 or above at the beginning of the cohort and had at least one year of up-to-standard pre-study follow-up. We used the CArdiovascular disease research using LInked Bespoke studies and Electronic health Records (CALIBER) dataset that links individuals in CPRD to national hospital admission and death records.[6] This linked dataset includes 4% of the English population and is broadly representative of the UK population in terms of age, sex, ethnicity and overall mortality.[7–9]

### Definition of dementia

We identified dementia in the three data sources using the clinical terms (Read version 2) (67 codes), ICD-9 (12 codes) and ICD-10 (36 codes) classification systems (Figure A and Table A in S1 File). We defined dementia as the record of one or more diagnostic codes in any of the three data sources at any time and in any position (i.e. dementia was any of the recorded diagnosis in hospital admission or death record). We defined people with corroborating evidence of diagnosis if they had dementia with: (i) more than one record of dementia in the same data source, on different dates; or (ii) a record of dementia in 2 or 3 data sources; or (iii) a record of falls, confusion, memory problems or nursing home admission; or (iv) dementia monitoring codes in primary care; or (v) a referral to a dementia speciality (geriatrics, care of the elderly, psychiatry); or (v) more than one prescription of rivastigmine, galantamine, donepezil, memantine, which are typically used to treat patients with Alzheimer’s disease and sometimes patients with dementia in Parkinson’s disease and dementia with Lewy bodies. We additionally classified people with dementia into four sub-types, Alzheimer’s disease, vascular, rare and unclassified; using Read and ICD diagnostic codes. Rare dementia included fronto-temporal dementia, dementia with Lewy bodies, Parkinson’s, Huntingdon’s, Pick’s or Creutzfeld-Jakob diseases, and HIV-related dementia.

### Selection of comparison group

For each case with dementia identified in primary or hospital care, we randomly selected up to ten people without dementia (concurrent sampling), who were matched on sex, year of birth and general practice. Controls had to be alive and actively registered in the general practice at the date of diagnosis of the matched dementia case, and to have had a contact with the practice within the year prior or after the matched index date. A total of 47151 people with dementia (99.5% of the total identified) were matched. They were followed up until death, transfer out of their primary care practice, or the date of administrative censoring (March 2010).

### Statistical analysis

We described the frequency and proportion of people with dementia and its subtypes, in the linked data and in each data source. We compared symptoms and management characteristics of people with dementia in cases and matched controls using conditional logistic regression that takes into account the matched structure of the data and consequently adjusts the results for the matching factors. We measured deprivation with the index of multiple deprivation, and divided the population into fifths based on this measure.[10] We calculated the 10-year and lifetime risks of dementia and Alzheimer’s according to age and gender, using Kaplan-Meier methods corrected for competing risk of death and using age as the time-scale. For this analysis we included all registered patients who were alive, registered in the cohort and without any dementia diagnosis, at the beginning of follow-up (i.e. earliest date of study eligibility), for example at their 65^th^, 75^th^ and 85^th^ birthdays. Such follow-up then ended on the date of first recorded dementia diagnosis (for cases) or the earliest of date of death, practice deregistration or last data collection date in the practice. We then estimated the point-prevalence of dementia in the entire cohort on 1^st^ July 2000 and 2005 and on 1^st^ January 2010. We counted as cases also patients who were diagnosed with dementia only at death, when this happened within a year from the analysis time points. Finally we compared overall and sex-specific mortality rates ratios in people with and without dementia in the matched subset. We performed this analysis using random-effects Poisson models, adjusted for age and sex as appropriate, with age as the time-scale. Cox proportional hazard models were not used because the hazard ratio for mortality was not constant over time. For this analysis the observation period began on the date of first recorded dementia diagnosis of the matched case.

All analyses were performed using Stata 13. The study was registered at www.clinicaltrials.gov (NCT02549872). Approval was granted by the Independent Scientific Advisory Committee of the Medicines and Healthcare products regulatory agency (protocol no. 15_138).

### Role of the funding source

The funding source had no role in the preparation of the manuscript.

## Results

### Diagnosis ascertainment and data source overlap

We identified 47,386 people with dementia, of whom 34,925 (74%) had corroborating evidence to support their diagnosis (Figure B in S1 File). A total of 22,184 (47%) could be classified into a dementia subtype: 12,633 (27%) had Alzheimer’s disease, 9,540 (20%) had vascular dementia and 1,539 (3%) had a rare dementia (Table B in S1 File). Compared to people with unclassified dementia, a greater proportion of those with a specific dementia subtype had corroborating evidence for their diagnosis (82% of Alzheimer’s disease, 71% of vascular, 69% of rare and 62% of unclassified subtype).

**Fig 1:**
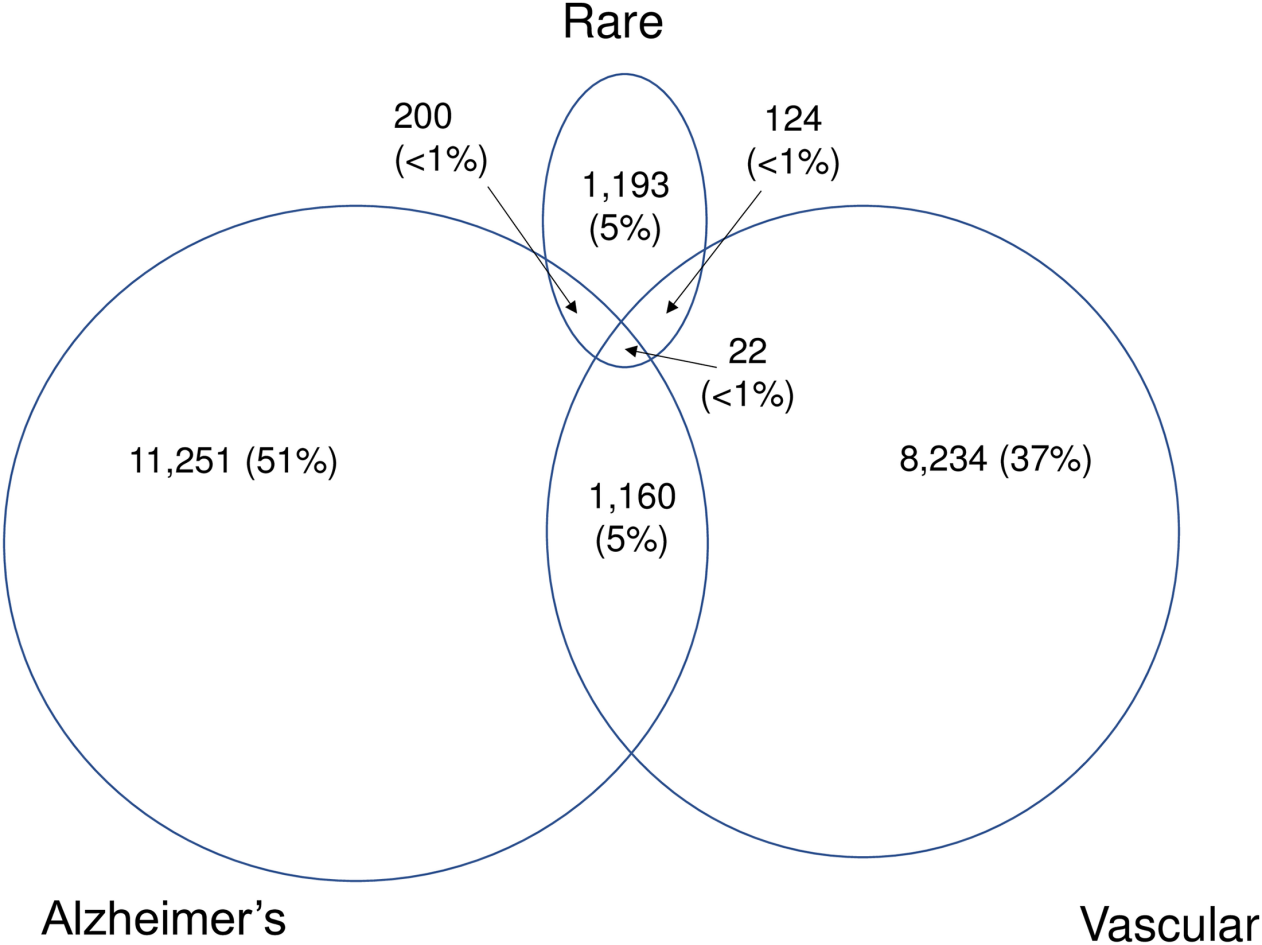
Capture of dementia in EHRs across the entire registration period in primary care, hospital episode statistics, and death records.

Of the 47,386 cases of dementia, 55% were captured in primary care, 65% in hospital records, and 26% in the national death register only (Table 1). Overall, 44% of dementia cases were captured in hospital or in the mortality registry but not in primary care, and 23% were captured in primary care records only (Fig 1). In each data source, most people had corroborating evidence of dementia (88% in primary care, 76% in hospital and 83% in the death registry). Overall, the proportion of people with dementia who had corroborating evidence was 74%. Compared with other data sources, a higher proportion dementia cases captured in the primary care were prescribed dementia medication (18% versus 11% in hospital or 9% in the mortality registry), had recorded symptoms (27% versus 23% or 25%) and had evidence of dementia monitoring (40% versus 22% or 22%) or of referral to a relevant speciality (12% versus 10% or 8%).

**Table 1:** Capture of diagnosis of dementia and overlap between data sources.

Dementia diagnosis	All	Primary care	Hospital	Mortality registry
**No of people**	47,386	26,269	31,034	12,232
**No. of people with corroborating evidence**	34,925 (74%)	23,225 (88%)	23,658 (76%)	10,191 (83%)
In multiple data sources	18,288 (39%)	15,348 (58%)	15,913 (51%)	9,176 (75%)
Multiple records in same data source	19,465 (41%)	8,770 (33%)	13,805 (44%)	NA
Prescribed dementia medication	5,264 (12%)	4,438 (18%)	3,087 (11%)	1,001 (9%)
Dementia symptoms	11,066 (23%)	7,096 (27%)	7,008 (23%)	3,075 (25%)
Dementia monitoring in primary care	12,590 (27%)	10,537 (40%)	6,977 (22%)	2,664 (22%)
Referral to relevant speciality	4,509 (10%)	3,038 (12%)	3,031 (10%)	963 (8%)

Note: NA, non-applicable

**Fig 2:**
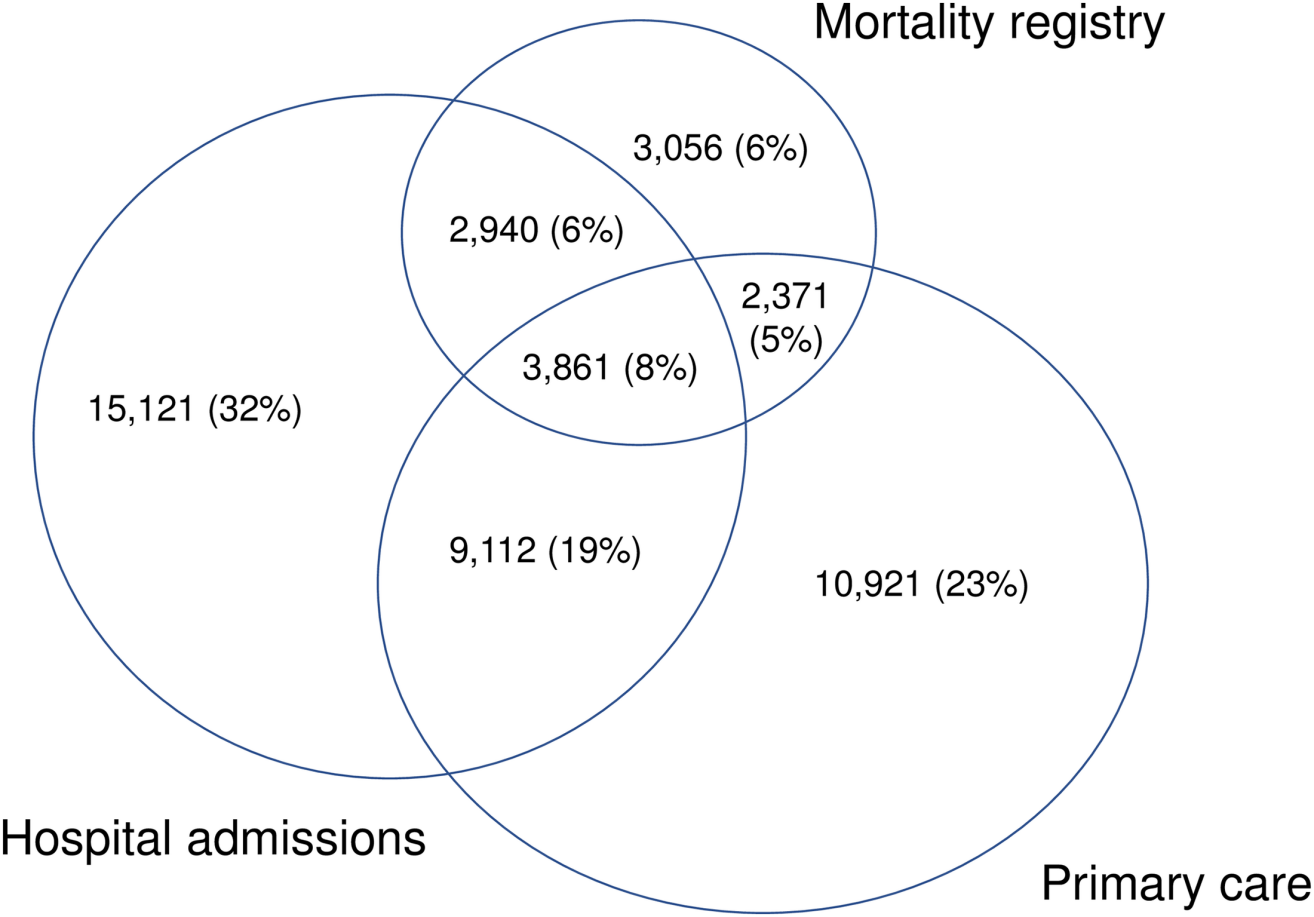
Capture of dementia by vascular dementia, Alzheimer’s dementia, rare dementia and dementia without specific diagnosis.

Overall, a minority of patients with dementia had a primary care record of memory impairment, confusion or admission to nursing home (23%), a record of dementia monitoring (27%) or had been referred to a geriatrician or care of the elderly psychiatrist (10%). Drugs typically indicated for Alzheimer’s dementia, dementia with Lewy bodies or dementia with Parkinson’s disease were more commonly prescribed to people with Alzheimer’s disease (27%) or rare (26%) dementias than to people with vascular (6%) or with unclassified dementia subtype (5%). Dementia subtypes were broadly consistently recorded across data sources, i.e. only 9% of people with Alzheimer’s disease also had vascular codes, and only 12% of those with vascular dementia also had Alzheimer’s codes; (Fig 2, Table B in S1 File).

There were 647 people with 2 or more prescriptions for dementia medication who had no dementia diagnosis in any of the three data sources. The characteristics of this group were similar to people identified with dementia i.e. 32% had dementia symptoms or nursing home admission, 14% were monitored for dementia in primary care, and 14% had been referred to a dementia relevant speciality.

### Factors associated with dementia

At diagnosis, most people with dementia were over 80 years old; 3% were <60 years, 6% were 60–69 years, 25% were 70–79 years and 66% were >80 years (Table C in S1 File). Sixty-six percent of them were women. Compared with matched controls, people with dementia were more likely to live in a deprived area (conditional OR 1.26, 95%CI: 1.20–1.31 for most vs least deprived; Table 2). They were also more likely to have record of memory impairment (conditional OR 11.97, 95%CI:11.24–12.75), confusion (conditional OR 9.57, 95%CI:9.14–10.02), falls (conditional OR 2.36 95%CI:2.31–2.41), depression (conditional OR 2.03, 95%CI:1.98–2.09) or anxiety (conditional OR 1.27, 95%CI:1.23–1.32). Where a diagnosis of depression was recorded, it was prior to dementia diagnosis in 71% of cases (5993/8449). People with dementia more often had a recorded power of attorney (conditional OR 8.34, 95%CI: 7.24–9.61), or had been admitted to a nursing home (conditional OR 7.09, 6.75–7.44).

**Table 2:** Characteristics of people with dementia compared with age-, practice- and sex-matched controls.

	People with dementia	People without dementia	Adjusted conditional OR (95%CI)
	N = 47,151	N = 324,627	
**Index of multiple deprivation (fifths)**			
1 (least)	10,741 (23%)	77,516 (24%)	1
2	10,860 (23%)	76,460 (24%)	1.05 (1.0–1.09)
3	9,986 (21%)	66,982 (21%)	1.12 (1.08–1.16)
4	8,440 (18%)	58,978 (18%)	1.12 (1.08–1.16)
5 (most)	7,124 (15%)	44,691 (14%)	1.26 (1.20–1.31)
**Memory impairment**			
Yes	2,769 (6%)	1,842 (1%)	11.97 (11.24–12.75)
No	47,382 (94%)	322,785 (99%)	1
**Confusion**			
Yes	4,829 (10%)	3,844 (1%)	9.57 (9.14–10.02)
No	42,322 (90%)	320,783 (99%)	1
**Falls**			
Yes	18,673 (40%)	67,268 (21%)	2.36 (2.31–2.41)
No	28,478 (60%)	257,359 (79%)	1
**Depression**			
Yes	8,449 (18%)	31,946 (10%)	2.03 (1.98–2.09)
No	38,702 (82%)	292,681 (90%)	1
**Anxiety**			
Yes	4,262 (9%)	24,046 (7%)	1.27 (1.23–1.32)
No	42,889 (91%)	300,581 (93%)	1
**Admission to nursing home**			
Yes	4,447 (9%)	4,868 (1%)	7.09 (6.75–7.44)
No	42,704 (91%)	319,759 (99%)	1
**Power of attorney**			
Yes	459 (1%)	362 (<1%)	8.34 (7.24–9.61)
No	46,692 (99%)	324,265 (>99%)	1
**Missed appointments**			
**Yes**	13,095 (28%)	87,548 (27%)	1.38 (1.11–1.16)
**No**	34,056 (72%)	237,079 (73%)	1
Median (IQR)^1^	1 (1–3)	1 (1–2)	
**GP consultation rate**^2^			
Missing	526 (1%)	0 (0%)	
0	23,993 (51%)	19,637 (6%)	1
0–5	5,564 (12%)	123,302 (38%)	0.04 (0.04–0.04)
>5	17,068 (36%)	181,688 (56%)	0.07 (0.07–0.07)
**Hospital admission rate**^2^			
Missing			
0	526 (1%)	0 (0%)	
0–1	16,828 (36%)	161,866 (50%)	1
1–2	28,412 (60%)	161,934 (50%)	1.67 (1.63–1.71)
>2	1,385 (3%)	827 (<1%)	15.15 (13.83–16.59)

^1^Amongst people with at least one missed appointment;

^2^Annual rate in the 5 years prior to entry

However only a minority of people with a diagnosis of dementia had a record of any one of these factors. The proportion of patients with dementia who had a missed a GP appointment was similar to patients without dementia (28% vs 27%, OR 1.38, 1.11–1.16), and the annual rate of GP consultation was similar in the two groups (proportions with >5 appointments per year were 36% vs 56%). However, the annual hospital admission rate was higher in people with than without dementia (proportions of >2 admissions per year were 3% vs <1%).

### Prevalence of dementia

**Fig 3:**
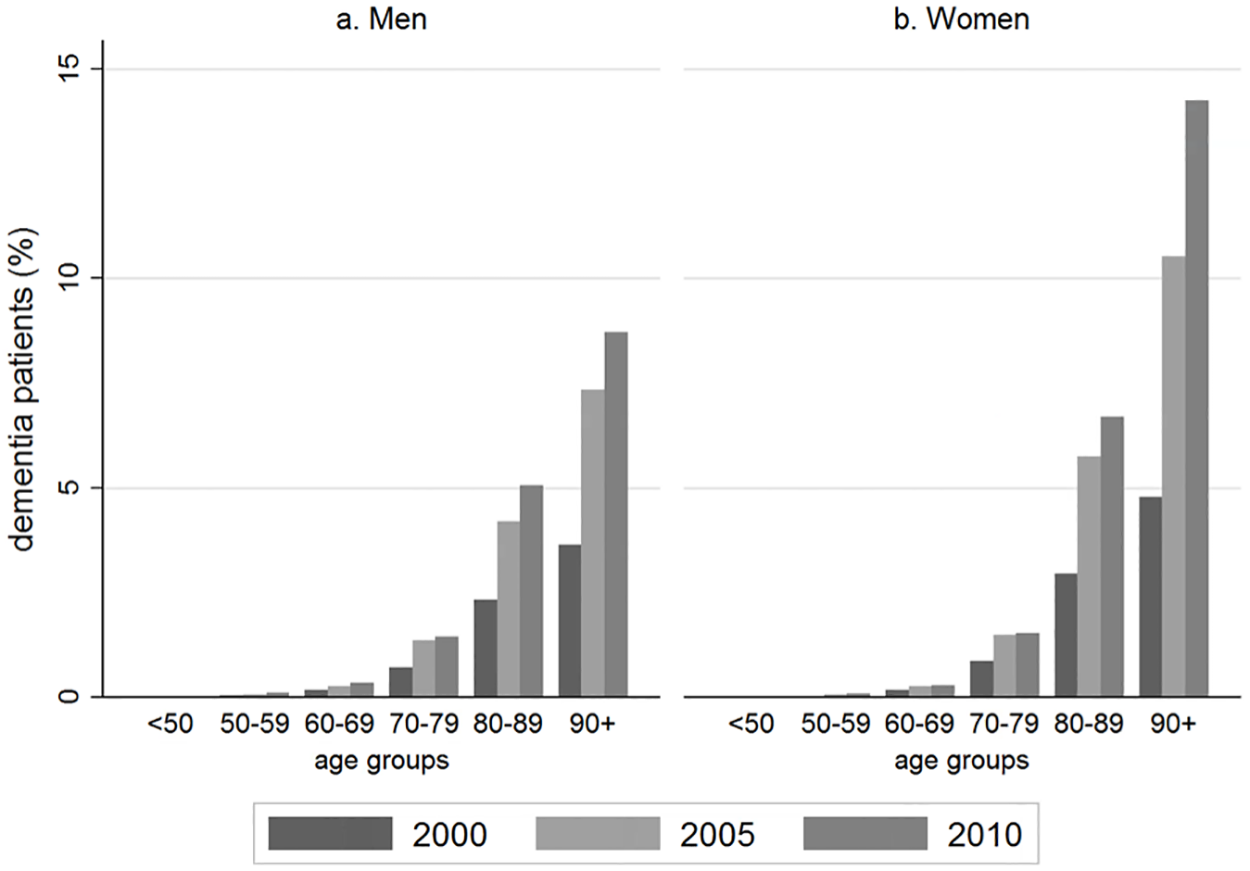
Time trends in prevalence of dementia according to age group and sex. Point prevalence estimated on 1^st^ July 2000 and 2005 and 1^st^ January 2010.

The prevalence of dementia increased markedly with age and over time, in both men and women (Fig 3). In 2010, in men the prevalence was much higher over 90 (8.7%) than under 50 years (0.2%) (Table C in S1 File). The estimates were substantially higher in women than in men in the eldest age group (14.2% vs. 8.7% in 2010). The prevalence of dementia gradually increased over time in men and women of all ages, for almost all dementia subtypes (Figure C in S1 File) and for diagnosis captured in primary and hospital records (Figure D in S1 File).

### Lifetime risks of dementia

Estimates of lifetime risk of dementia and Alzheimer’s disease are shown in Table 3. The lifetime risk of dementia in women increased modestly with age, from 14.9% (95%CI 14.7%-15.1%) at 65 years to 21.8% (95%CI 21.3%-22.3%) at 85 years. Lower estimates were found in men: 9.2% (95%CI 9.0%-9.4%) at 65 years, and 15.4% (95%CI 14.7%-16.0%) at 85 years.

**Table 3:** Lifetime risk of dementia in men and women estimated in linked primary care, hospital episode and death records.

	All dementia			Alzheimer’s disease		
	No. of people at risk	10-year risk in %	Lifetime risk in %*	No. of people at risk	10-year risk in %	Lifetime risk in %*
**Women**						
**65 years**	321,851	2.8 (2.7–2.9)	14.9 (14.7–15.1)	321,851	1.0 (0.9–1.1)	4.9 (4.7–5.0)
**75 years**	197,918	16.6 (16.3–16.8)	21.7 (21.4–21.9)	197,918	5.0 (4.8–5.1)	6.2 (6.0–6.3)
**85 years**	83,694	23.8 (23.3–24.3)	21.8 (21.3–22.3)	83,694	4.5 (4.2–4.7)	4.1 (3.9–4.4)
**Men**						
**65 years**	253,203	2.7 (2.6–2.8)	9.2 (9.0–9.4)	253,203	0.7 (0.7–0.8)	2.5 (2.4–2.6)
**75 years**	128,957	12.8 (12.6–13.1)	14.5 (14.2–14.7)	128,957	3.4 (3.2–3.5)	3.7 (3.5–3.8)
**85 years**	38,822	17.4 (16.7–18.2)	15.4 (14.7–16.0)	38,822	3.5 (3.1–3.8)	3.2 (2.9–3.5)

Note:

(*) Lifetime risk calculated on the median residual life from the UK National Lifetable. For women aged 65, 75 and 85 the median years of residual life expectancy were 21.04, 13.11 and 6.81 years respectively, whereas for men aged 65, 75 and 85 they were 18.61, 11.35 and 5.85 years respectively.

### Mortality associated with dementia

In total, 159,674 deaths were recorded during a median follow-up of 1.7 years (inter-quartile range 0.6 to 3.4), 34,528 amongst people with dementia and 125,146 amongst those without dementia (Table 4). The incidence rate ratio of mortality for people with dementia compared to individuals without the disease was 1.56 (95% CI:1.54–1.58). Estimates were similar in men and in women.

**Table 4:** Association between dementia and mortality.

	No. of people	No. of deaths	IRR (95% CI)
**Overall**			
People without dementia	324,627	125,146	1
People with dementia	47,151	34,528	1.56 (1.54–1.58)
**Men**			
People without dementia	132,251	51,882	1
People with dementia	16,088	11,982	1.61 (1.58–1.64)
**Women**			
People without dementia	192,376	73,264	1
People with dementia	31,063	22,546	1.53 (1.51–1.56)

Note: IRR, incidence rate ratios adjusted for age and sex (as appropriate) from random-effects Poisson models comparing people with dementia and randomly selected people without dementia matched for age, sex and general practice

## Discussion

Using contemporary, nationally-representative linked primary care, hospital records and the death registry from 2,524,144 people in England and Wales, we identified people with recorded diagnostic codes of dementia and dementia subtypes. The large majority of people with dementia had corroborating evidence of diagnosis, including recording of multiple diagnostic records in one or more data sources, symptoms and care features characteristics of dementia or were prescribed dementia medication. The lifetime risk of dementia at 65 years was 15% in women and 9% in men, and mortality was 1.56 higher in people with than without dementia.

Our findings highlight the importance of using multiple linked data sources for defining dementia in EHRs. No individual data source analysed had complete coverage of coded dementia. Six percent were only recorded in the death registry, thirty-two percent only in hospital records and twenty-three percent only in primary care. Because data in CALIBER were anonymised, we could not validate dementia cases against patient clinical charts.

However, subgroups of dementia codes have been validated in previous EHR-based studies.[4,5,11,12] In addition, our estimated dementia lifetime risks are similar to figures reported in previous population based cohort studies.[13] Our study suggests that Read-coded symptoms on their own, cannot be used to identify unrecorded patients with dementia, because these are infrequently recorded and are insufficiently specific, even in combination, to accurately identify cases. Future work with natural language processing methods of free text collected during the consultation would be needed to make better use of symptom data in electronic records.

In studies based on the analysis of EHRs, lifetime risk of dementia is likely to be a more suitable measure of disease risk than absolute incidence rates, given that the time of onset of dementia is difficult to define in clinical practice. Lifetime risk is probably the most important statistic for an individual when planning their future needs. The lifetime risk of dementia depends on the duration of life, and is affected by the competing risks of death from other causes and the incidence of dementia in a population. We compared the 10 year risk of dementia from the Framingham study [14] with our estimates (Table E in S1 File). Our lifetime risk estimates were slightly higher than those reported in the US in 2005. For example, the 10 year risks of all dementia found in the Framingham study at 75 in men was 7.6%, and in women 7.4%, as compared with 12.8% and 16.6% in our study.

The prevalence of dementia at different ages in this study in England was lower than estimates reported in a community prevalence study with participants recruited from Cambridgeshire, Liverpool and Newcastle. In that study in 2001, prevalences from age 65–69, 70–74, 75–79, 80–84, 85–90 and over 90 in men were: 1.2, 3.0, 5.2, 10.6, 12.8, 17.1% and in women 1.8, 2.5, 6.2, 9.5, 18.1, and 35%. Our prevalence estimates were approximately half of this in 2000, but about two-thirds of this in 2010, perhaps indicating improvement in recording. Community based incidence studies using formal instruments are likely to ascertain dementia with a lower severity, and it is possible that those in the EHRs represent only those in a later stage of illness, those with clinically evident dementia [15] or with more severe symptoms.[12] Given that the benefits of early dementia diagnosis have not been shown, and that the impact that such diagnosis has on patients and their relatives, GPs may hesitate to formally diagnose the disease until symptoms become disabling.

Although we have examined UK linked EHR from 1998 to 2010, our conclusions may not be transportable to other EHR datasets covering different time periods. With changing UK practice, diagnostic validity of a dementia record may change, with better ascertainment achieved in recent years after a significant effort to improve dementia diagnosis in primary care. Our results are not generalizable to other health systems, and therefore researchers working with data from these systems should aim to determine the validity of dementia diagnosis, either by linking and/or comparing information from existing or new disease cohorts to their EHR data sources, though case review, or conducting a similar analysis to ours.[16]

Although linked EHR are an efficient source of data for dementia research, there are a number of weakness to be considered. First, there may be variation in case ascertainment and validity of diagnosis across regions, depending on hospital or primary care diagnostic or management behaviours. Second, under ascertainment of diagnosis during the early stages of the condition is likely- hence our recommendation for a lifetime approach. Finally, there is often limited data on important prognostic factors, such as education and family history or APOE4 allele, although these might eventually be obtained through linkage to other data sources.

## Conclusion

This study provides evidence that the diagnosis of dementia in linked electronic health records has sufficient validity for large scale epidemiological studies. The major value of current records is found in coded diagnoses, rather than additional symptoms or other care episodes, which are seldom recorded. Despite reasonable concerns that that electronic health records underestimate the point prevalence of dementia compared to research studies, the calculated lifetime risk of dementia from these electronic health records is similar to population based estimates.

## Supporting information

S1 File Supplementary data. (Table A) Dementia diagnostic codes used to identify people with dementia in hospital (ICD-10), mortality registry (ICD-9 and ICD-10) and primary care (Read codes). (Table B) Capture of diagnosis of dementia and its subtypes and overlap between data sources. (Table C) Time trends in prevalence of dementia according to age group and sex. (Table D) Median residual life expectancy in years. (Table E) Comparison of 10-year lifetime risks of dementia in our study and in the Framingham study. (Figure A) Defining cases of dementia and non-dementia in national samples of structured electronic health records: phenotype algorithm using multiple ontologies (ICD-10, Read-2, BNF). (Figure B) Study flow chart. (Figure C) Time trends in prevalence of dementia subtypes according to age group. (Figure D) Time trends in prevalence of dementia diagnosis according to data source capture.
(DOCX)
