# Correction: The diagnosis, burden and prognosis of dementia: A record-linkage cohort study in England

**DOI:** 10.1371/journal.pone.0201213

**Published:** 2018-07-19

**Authors:** Mar Pujades-Rodriguez, Valentina Assi, Arturo Gonzalez-Izquierdo, Tim Wilkinson, Christian Schnier, Cathie Sudlow, Harry Hemingway, William N. Whiteley

The images for Figs 1 and 2 are incorrectly switched. The image that appears as Fig 1 should be Fig 2, and the image that appears as Fig 2 should be Fig 1. The figure captions appear in the correct order.

**Fig 1:**
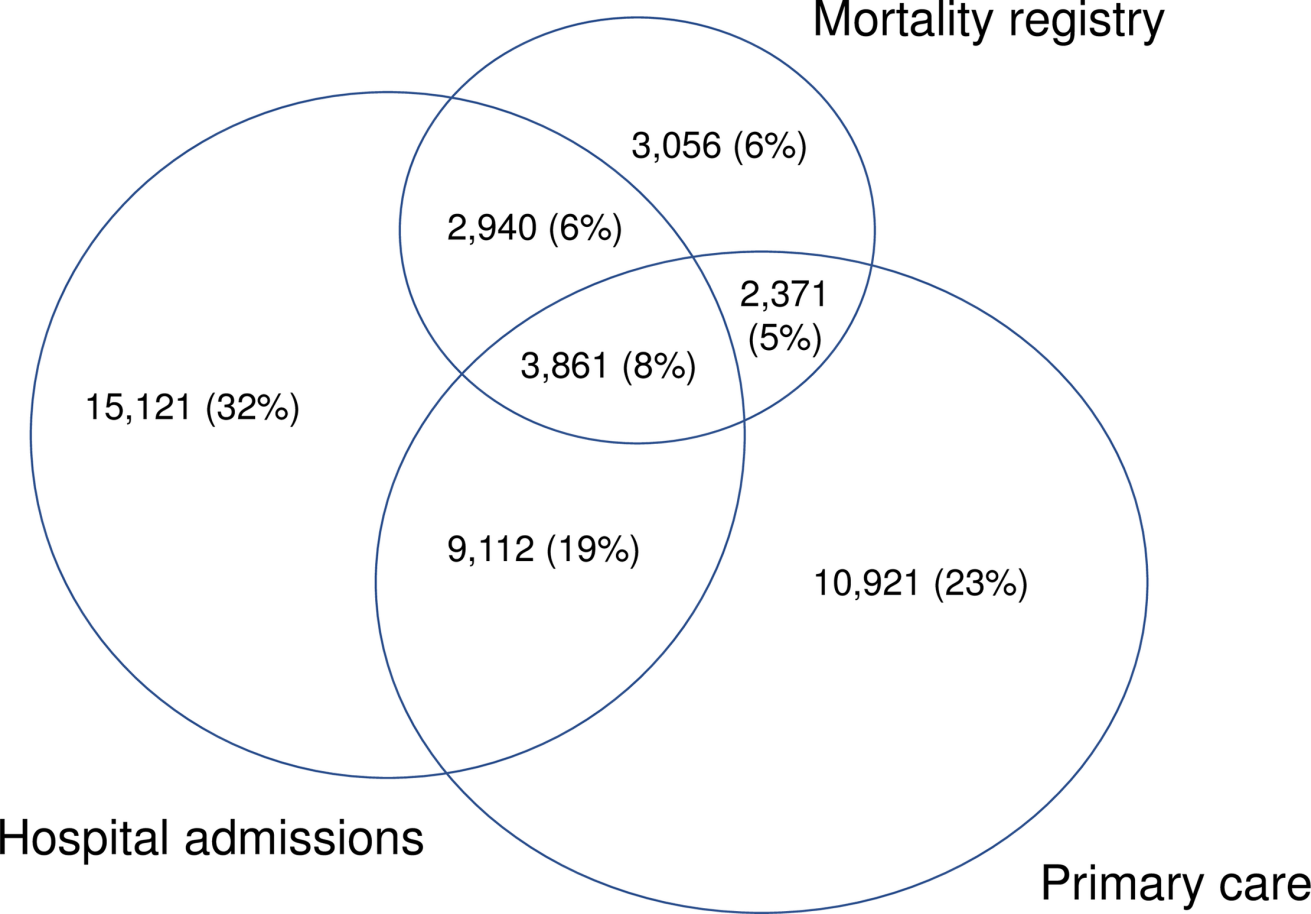
Capture of dementia in EHRs across the entire registration period in primary care, hospital episode statistics, and death records.

**Fig 2:**
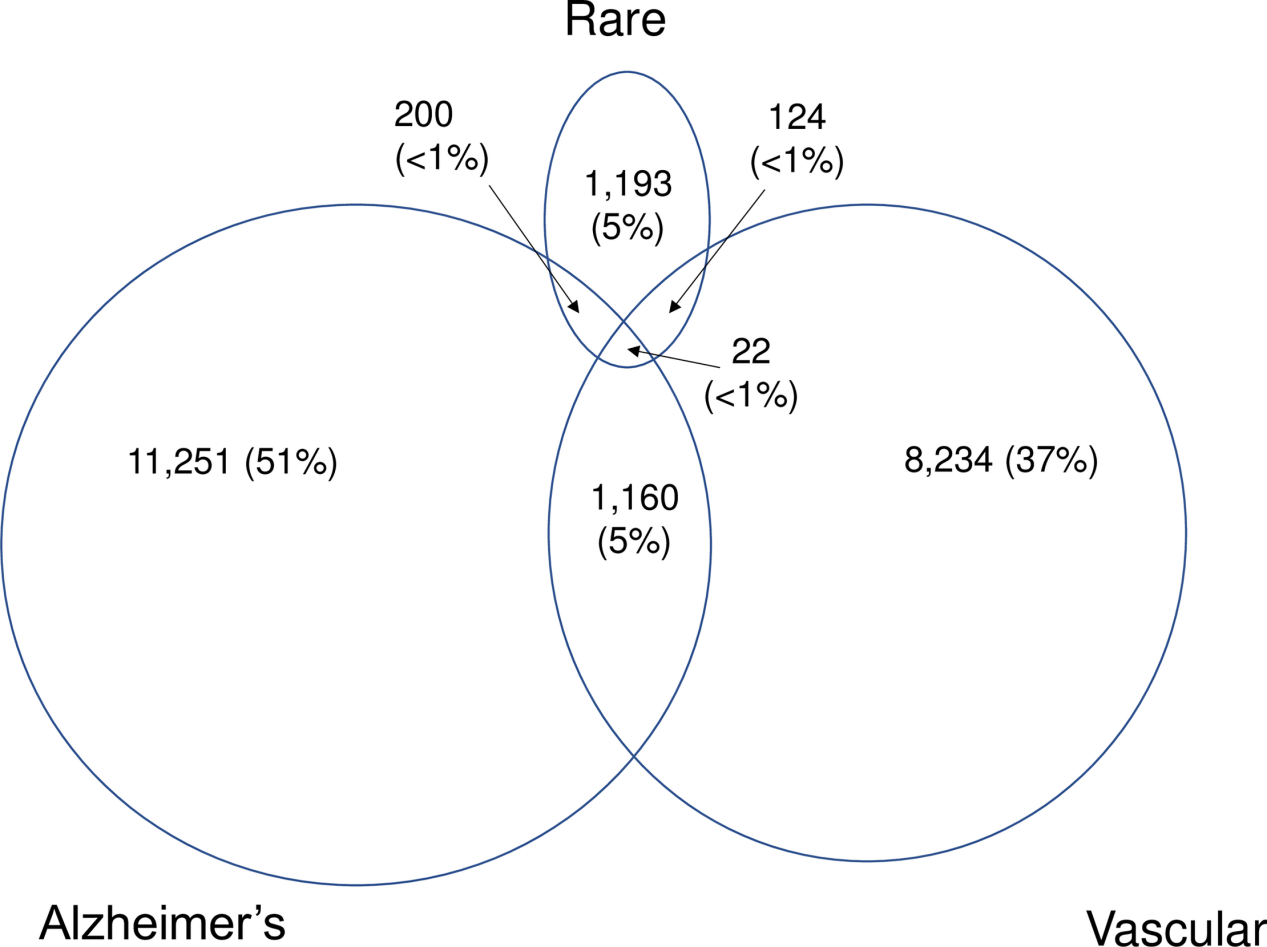
Capture of dementia by vascular dementia, Alzheimer’s dementia, rare dementia and dementia without specific diagnosis.
