# Supplementary material for: The diagnosis, burden and prognosis of dementia: A record-linkage cohort study in England
Source: PLoS One. 2018 Jun 26;13(6):e0199026. doi: 10.1371/journal.pone.0199026 (PMC6019102; doi:10.1371/journal.pone.0199026)
Supplement: S1 File — (Table A) Dementia diagnostic codes used to identify people with dementia in hospital (ICD-10), mortality registry (ICD-9 and ICD-10) and primary care (Read codes). (Table B) Capture of diagnosis of dementia and its subtypes and overlap between data sources. (Table C) Time trends in prevalence of dementia according to age group and sex. (Table D) Median residual life expectancy in years. (Table E) Comparison of 10-year lifetime risks of dementia in our study and in the Framingham study. (Figure A) Defining cases of dementia and non-dementia in national samples of structured electronic health records: phenotype algorithm using multiple ontologies (ICD-10, Read-2, BNF). (Figure B) Study flow chart. (Figure C) Time trends in prevalence of dementia subtypes according to age group. (Figure D) Time trends in prevalence of dementia diagnosis according to data source capture. (DOCX) [file pone.0199026.s001.docx]

**SUPPLEMENTARY MATERIAL**

**TABLES**

Table A. Dementia diagnostic codes used to identify people with dementia in hospital (ICD-10), mortality registry (ICD-9 and ICD-10) and primary care (Read codes)

Table B. Capture of diagnosis of dementia and its subtypes and overlap between data sources

Table C. Time trends in prevalence of dementia according to age group and sex

Table D. Median residual life expectancy in years

Table E. Comparison of 10-year lifetime risks of dementia in our study and in the Framingham study

**FIGURES**

Figure A. Defining cases of dementia and non-dementia in national samples of structured electronic health records: phenotype algorithm using multiple ontologies (ICD-10, Read-2, BNF)

Figure B. Study flow chart

Figure C. Time trends in prevalence of dementia subtypes according to age group

Figure D. Time trends in prevalence of dementia diagnosis according to data source capture

**Table A. Dementia diagnostic codes used to identify people with dementia in hospital (ICD-10), mortality registry (ICD-9 and ICD-10) and primary care (Read codes)**

| **ICD-10** |  | | | |
| --- | --- | --- | --- | --- |
|  | **Alzheimer’s disease** | | | |
| F00 | Dementia in Alzheimer's disease | | | |
| F000A | Dementia in Alzheimer's disease with early onset | | | |
| F001A | Dementia in Alzheimer's disease with late onset | | | |
| F002A | Dementia in Alzheimer's disease, atypical or mixed type | | | |
| F009A | Dementia in Alzheimer's disease, unspecified | | | |
| G30 | Alzheimer’s disease | | | |
| G30.0 | Alzheimer’s disease with early onset | | | |
| G30.1 | Alzheimer’s disease with late onset | | | |
| G30.8 | Other Alzheimer disease, unspecified | | | |
| G30.9 | Alzheimer's disease unspecified | | | |
|  | **Vascular dementia** | | | |
| F01 | Vascular dementia | | | |
| F010 | Vascular dementia of acute onset | | | |
| F011 | Multi-infarct dementia | | | |
| F012 | Subcortical vascular dementia | | | |
| F013 | Mixed cortical and subcortical vascular dementia | | | |
| F018 | Other vascular dementia | | | |
| F019 | Vascular dementia, unspecified | | | |
| I67.3 | Binswanger's disease | | | |
|  | **Rare dementia** | | | |
| F020A | Dementia in Pick's disease | | | |
| F021A | Dementia in Creutzfeldt-Jakob disease | | | |
| F022A | Dementia in Huntington's disease | | | |
| F023A | Dementia in Parkinson's disease | | | |
| F024A | Dementia in human immunodef virus [HIV] disease | | | |
| F028A | Dementia in other specified diseases classified elsewhere | | | |
|  | **Unspecified dementia type** | | | |
| F02 | Dementia in other diseases classified elsewhere | | | |
| F03X | Unspecified dementia | | | |
| F051 | Delirium superimposed on dementia | | | |
|  | **Possible dementia** | | | |
| F050 | Delirium not superimposed on dementia, so described | | | |
| G31.0 | Circumscribed brain atrophy | | | |
| G31.1 | Senile degeneration of brain, not otherwise classified | | | |
| G31.2 | Degeneration of nervous system due to alcohol | | | |
| G31.8 | Other specified degenerative diseases of nervous system | | | |
| G31.9 | Degenerative disease of nervous system, unspecified | | | |
| **ICD-9** |  | | | |
|  | **Alzheimer’s disease** | | | |
| 331.0 | Alzheimer's disease | | | |
|  | **Vascular dementia** | | | |
| 290.4 | Vascular dementia | | | |
|  | **Rare dementia** | | | |
| 046.19 | Creutzfeld Jacob | | | |
| 333.4 | Huntingdon's | | | |
| 331.1 | Frontotemporal dementia | | | |
|  | **Unspecified dementia type** | | | |
| 290.0 | Senile dementia, uncomplicated | | | |
| 290.1x | Presenile dementia | | | |
| 290.2 | Senile dementia with delusional features | | | |
| 290.3 | Senile dementia with delirium | | | |
|  | **Possible dementia** | | | |
| 294.9 | unspecified persistent mental disorders | | | |
| 331.2 | Senile degeneration | | | |
| 331.9 | Cerebral degeneration unspecified | | | |
|  | |  | |  |
| **Read code** | |  | |  |
|  | **Alzheimer’s disease** | |  |  |
| Eu00012 | [X]Primary degen dementia, Alzheimer's type, presenile onset | |  |  |
| Eu00013 | [X]Alzheimer's disease type 2 | |  |  |
| Fyu3000 | [X]Other Alzheimer's disease | |  |  |
| Eu00111 | [X]Alzheimer's disease type 1 | |  |  |
| Eu01111 | [X]Predominantly cortical dementia | |  |  |
| Eu00113 | [X]Primary degen dementia of Alzheimer's type, senile onset | |  |  |
| Eu00011 | [X]Presenile dementia,Alzheimer's type | |  |  |
| Eu00000 | [X]Dementia in Alzheimer's disease with early onset | |  |  |
| Eu00z00 | [X]Dementia in Alzheimer's disease, unspecified | |  |  |
| Eu00100 | [X]Dementia in Alzheimer's disease with late onset | |  |  |
| F110100 | Alzheimer's disease with late onset | |  |  |
| F110000 | Alzheimer's disease with early onset | |  |  |
| Eu00200 | [X]Dementia in Alzheimer's dis, atypical or mixed type | |  |  |
| Eu00112 | [X]Senile dementia,Alzheimer's type | |  |  |
| Eu00z11 | [X]Alzheimer's dementia unspec | |  |  |
| Eu00.00 | [X]Dementia in Alzheimer's disease | |  |  |
| F110.00 | Alzheimer's disease | |  |  |
|  | **Vascular dementia** | |  |  |
| E004100 | Arteriosclerotic dementia with delirium | |  |  |
| Eu01000 | [X]Vascular dementia of acute onset | |  |  |
| E004200 | Arteriosclerotic dementia with paranoia | |  |  |
| Eu01y00 | [X]Other vascular dementia | |  |  |
| E004300 | Arteriosclerotic dementia with depression | |  |  |
| Eu01200 | [X]Subcortical vascular dementia | |  |  |
| E004000 | Uncomplicated arteriosclerotic dementia | |  |  |
| Eu01300 | [X]Mixed cortical and subcortical vascular dementia | |  |  |
| Eu01z00 | [X]Vascular dementia, unspecified | |  |  |
| E004z00 | Arteriosclerotic dementia NOS | |  |  |
| Eu01.11 | [X]Arteriosclerotic dementia | |  |  |
| Eu01100 | [X]Multi-infarct dementia | |  |  |
| E004.00 | Arteriosclerotic dementia | |  |  |
| E004.11 | Multi infarct dementia | |  |  |
| Eu01.00 | [X]Vascular dementia | |  |  |
|  | **Rare dementia** | |  |  |
| Eu02400 | [X]Dementia in human immunodef virus [HIV] disease | |  |  |
| Eu02100 | [X]Dementia in Creutzfeldt-Jakob disease | |  |  |
| Eu02200 | [X]Dementia in Huntington's disease | |  |  |
| Eu02000 | [X]Dementia in Pick's disease | |  |  |
| F111.00 | Pick's disease | |  |  |
| Eu02300 | [X]Dementia in Parkinson's disease | |  |  |
| Eu02500 | [X]Lewy body dementia | |  |  |
| F116.00 | Lewy body disease | |  |  |
|  | **Unspecified dementia type** | |  |  |
| E002z00 | Senile dementia with depressive or paranoid features NOS | |  |  |
| Eu02z11 | [X] Presenile dementia NOS | |  |  |
| Eu02y00 | [X]Dementia in other specified diseases classif elsewhere | |  |  |
| E001000 | Uncomplicated presenile dementia | |  |  |
| E001100 | Presenile dementia with delirium | |  |  |
| E012.00 | Other alcoholic dementia | |  |  |
| Eu04100 | [X]Delirium superimposed on dementia | |  |  |
| Eu02z13 | [X] Primary degenerative dementia NOS | |  |  |
| E001300 | Presenile dementia with depression | |  |  |
| E001200 | Presenile dementia with paranoia | |  |  |
| Eu02z16 | [X] Senile dementia, depressed or paranoid type | |  |  |
| E001z00 | Presenile dementia NOS | |  |  |
| E002.00 | Senile dementia with depressive or paranoid features | |  |  |
| E003.00 | Senile dementia with delirium | |  |  |
| Eu10711 | [X]Alcoholic dementia NOS | |  |  |
| E012.11 | Alcoholic dementia NOS | |  |  |
| E041.00 | Dementia in conditions EC | |  |  |
| E002000 | Senile dementia with paranoia | |  |  |
| Eu02.00 | [X]Dementia in other diseases classified elsewhere | |  |  |
| E002100 | Senile dementia with depression | |  |  |
| E001.00 | Presenile dementia | |  |  |
| 9hD0.00 | Excepted from dementia quality indicators: Patient unsuitabl | |  |  |
| Eu02z14 | [X] Senile dementia NOS | |  |  |
| E000.00 | Uncomplicated senile dementia | |  |  |
| Eu02z00 | [X] Unspecified dementia | |  |  |
| E00..11 | Senile dementia | |  |  |
| E00..12 | Senile/presenile dementia | |  |  |
| E02y100 | Drug-induced dementia | |  |  |
|  | **Possible dementia** | |  |  |
| Eu04000 | [X]Delirium not superimposed on dementia, so described | |  |  |
|  |  | |  |  |

**Note:** Possible dementia codes were used for exclusion from the potentially eligible individuals for the random selection of people without dementia (matched sample)

**Table B. Capture of diagnosis of dementia and its subtypes and overlap between data sources**

|  | **All** | **Unclassified** | **Alzheimer’s** | **Vascular** | **Rare** |
| --- | --- | --- | --- | --- | --- |
| **No. of people with dementia diagnosis** | 47386 | 25208 | 12633 | 9540 | 1539 |
| **No. of people with dementia diagnosis and corroborating evidence** | 34925 (74%) | 15523 (62%) | 10296 (82%) | 6735 (71%) | 1065 (70%) |
| Recorded in multiple data sources* | 18288 (39%) | 6437 (26%) | 3634 (29%) | 1919 (20%) | 208 (14%) |
| Multiple records in same data source* | 19465 (41%) | 6735 (27%) | 5388 (43%) | 2507 (26%) | 395 (26%) |
| Prescribed dementia medication | 5264 (12%) | 1284 (5%) | 3360 (27%) | 603 (6%) | 396 (26%) |
| Dementia symptoms | 11066 (23%) | 5203 (21%) | 3456 (27%) | 2515 (26%) | 380 (25%) |
| Dementia monitoring in primary care | 12590 (27%) | 4546 (18%) | 5001 (40%) | 3225 (34%) | 490 (32%) |
| Referral to relevant speciality | 4509 (10%) | 1883 (7%) | 1483 (12%) | 1185 (12%) | 184 (12%) |
|  |  |  |  |  |  |
| **Dementia subtype** |  |  |  |  |  |
| Alzheimer’s | 12633 (27%) | NA | - | 1182 (12%) | 222 (14%) |
| Vascular | 9540 (20%) | NA | 1182 (9%) | - | 146 (9%) |
| Rare | 1539 (3%) | NA | 222 (2%) | 146 (2%) | - |

*For columns related to each of the dementia subtypes, this refers to the number of people who had a diagnosis of the dementia subtype in more than one data source (e.g. in primary care and hospital) or in the same data source, as appropriate.

**Table C. Time trends in prevalence of dementia according to age group and sex**

|  |  | **Men** |  |  | **Women** |  |
| --- | --- | --- | --- | --- | --- | --- |
| **Age group** | **No. of people with dementia** | **Total population** | **Prevalence (%)** | **No. of people with dementia** | **Total population** | **Prevalence (%)** |
| **2000** |  |  |  |  |  |  |
| 18-49 | 11 | 342191 | <.01 | 16 | 330915 | <.04 |
| 50-59 | 40 | 107298 | 0.04 | 29 | 104566 | 0.03 |
| 60-69 | 130 | 74982 | 0.17 | 130 | 77244 | 0.17 |
| 70-79 | 381 | 54212 | 0.70 | 593 | 69091 | 0.86 |
| 80-89 | 501 | 21516 | 2.33 | 1171 | 39672 | 2.95 |
| 90+ | 105 | 2893 | 3.63 | 445 | 9318 | 4.78 |
| **2005** |  |  |  |  |  |  |
| 18-49 | 51 | 407168 | 0.01 | 36 | 392112 | 0.01 |
| 50-59 | 70 | 123416 | 0.06 | 83 | 121483 | 0.07 |
| 60-69 | 238 | 92079 | 0.26 | 234 | 93421 | 0.25 |
| 70-79 | 826 | 60834 | 1.36 | 1084 | 73490 | 1.48 |
| 80-89 | 1146 | 27274 | 4.20 | 2699 | 46995 | 5.74 |
| 90+ | 280 | 3816 | 7.34 | 1200 | 11408 | 10.52 |
| **2010** |  |  |  |  |  |  |
| 18-49 | 72 | 383801 | 0.02 | 61 | 372857 | 0.02 |
| 50-59 | 141 | 123930 | 0.11 | 109 | 120695 | 0.09 |
| 60-69 | 369 | 108741 | 0.34 | 313 | 110851 | 0.28 |
| 70-79 | 957 | 66187 | 1.45 | 1145 | 75422 | 1.52 |
| 80-89 | 1638 | 32406 | 5.05 | 3314 | 49468 | 6.70 |
| 90+ | 443 | 5087 | 8.71 | 1893 | 13295 | 14.24 |

Figure note: Point prevalence estimated on 1^st^ July 2000 and 2005 and 1^st^ January 2010

**Table D. Median residual life expectancy in years**

| **At age** | **Women** | **Men** |
| --- | --- | --- |
| **65** | 21.0 | 18.6 |
| **75** | 13.1 | 11.4 |
| **85** | 6.8 | 5.9 |

Note: Lifetime risk calculated on the median residual life from the UK National Lifetable. For women aged 65, 75 and 85 the median years of residual life expectancy were 21.04, 13.11 and 6.81 years respectively, whereas for men aged 65, 75 and 85 they were 18.61, 11.35 and 5.85 years respectively.

**Table E. Comparison of 10-year lifetime risks of dementia in our study and in the Framingham study**

|  | **10-year risk of dementia in our study in % (95% CI)** | **10-year risk of dementia in the Framingham study in % (95% CI)*** |
| --- | --- | --- |
| **Women** |  |  |
| **65** | 2.8 (2.7–2.9) | 1.0 (0.4-1.5) |
| **75** | 16.6 (16.3–16.8) | 7.4 (6.0-8.8) |
| **85** | 23.8 (23.3–24.3) | 20.3 (17.2-23.5) |
| **Men** |  |  |
| **65** | 2.7 (2.6–2.8) | 1.6 (0.8-2.4) |
| **75** | 12.8 (12.6–13.1) | 7.6 (5.8-9.3) |
| **85** | 17.4 (16.7-18.2) | 13.8 (9.9-17.7) |

*****Reported by Seshadri S, Beiser A, Kelly-Hayes M, *et al.* (2006) The Lifetime Risk of Stroke. *Stroke*; 37(2): 345-50.

**Figure A. Defining cases of dementia and non dementia in national samples of structured electronic health records: phenotype algorithm using multiple ontologies (ICD-10, Read-2, BNF)**


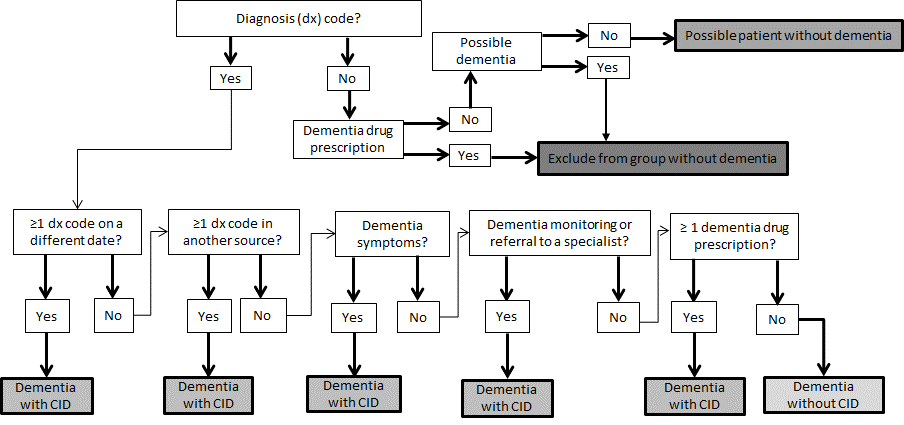


Note: CID, corroborating information for dementia diagnosis; dx, diagnostic. The phenotype algorithm uses multiple ontologies: the International Classification of Disease version 10 (ICD-10), version 2 of the Read classification system and the British National Formulary (BNF). CID includes multiple diagnostic codes recorded on different dates in the same data source, recorded diagnostic codes in 2 or 3 different data sources (e.g. primary care and hospital admissions), record of dementia symptoms (falls, confusion, cognitive deficiency or nursing home admission), record of dementia monitoring or referral to a dementia specialist (geriatrics, care of the elderly, psychiatry), or more than one prescription of dementia medication (rivastigmine, galantamine, donepezil, memantine) during the study period. The group of people with ‘possible dementia’ included those without diagnostic codes for dementia and with recorded unspecific codes listed in Table S1.

**Figure B. Study flowchart**

**No. of CALIBER people eligible**

(N=2,524,144)

**No. of people with dementia** (N=47,386)*

- Alzheimer’s: 12,633
- Vascular: 9,540
- Rare: 1,539
- Unclassified: 25,202

**No. of people with corroborating diagnosis evidence** (N=34,925)*

- Alzheimer’s: 10,296 (82%)
- Vascular: 6,735 (71%)
- Rare: 1,065 (69%)
- Unclassified: 15,519 (62%)

**No. of matched controls**

(N=324,627)

**No. of matched cases**

(N=47,151)

*Some people had more than one subtype of dementia

**Figure C. Time trends in prevalence of dementia subtypes according to age group**

**
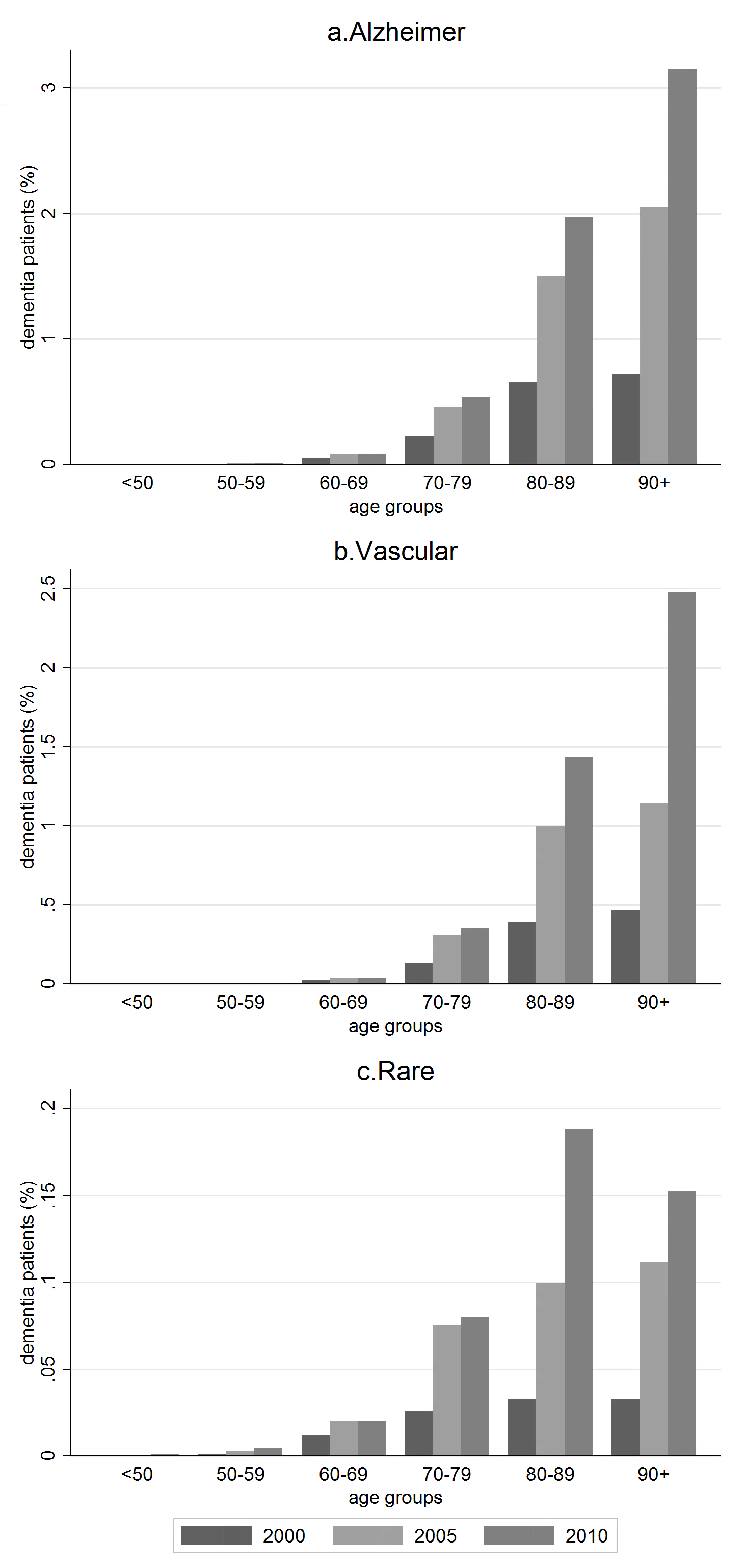
**

Figure note: Point prevalence estimated on 1^st^ July 2000 and 2005 and 1^st^ January 2010

**Figure D. Time trends in prevalence of dementia diagnosis according to data source capture**

**
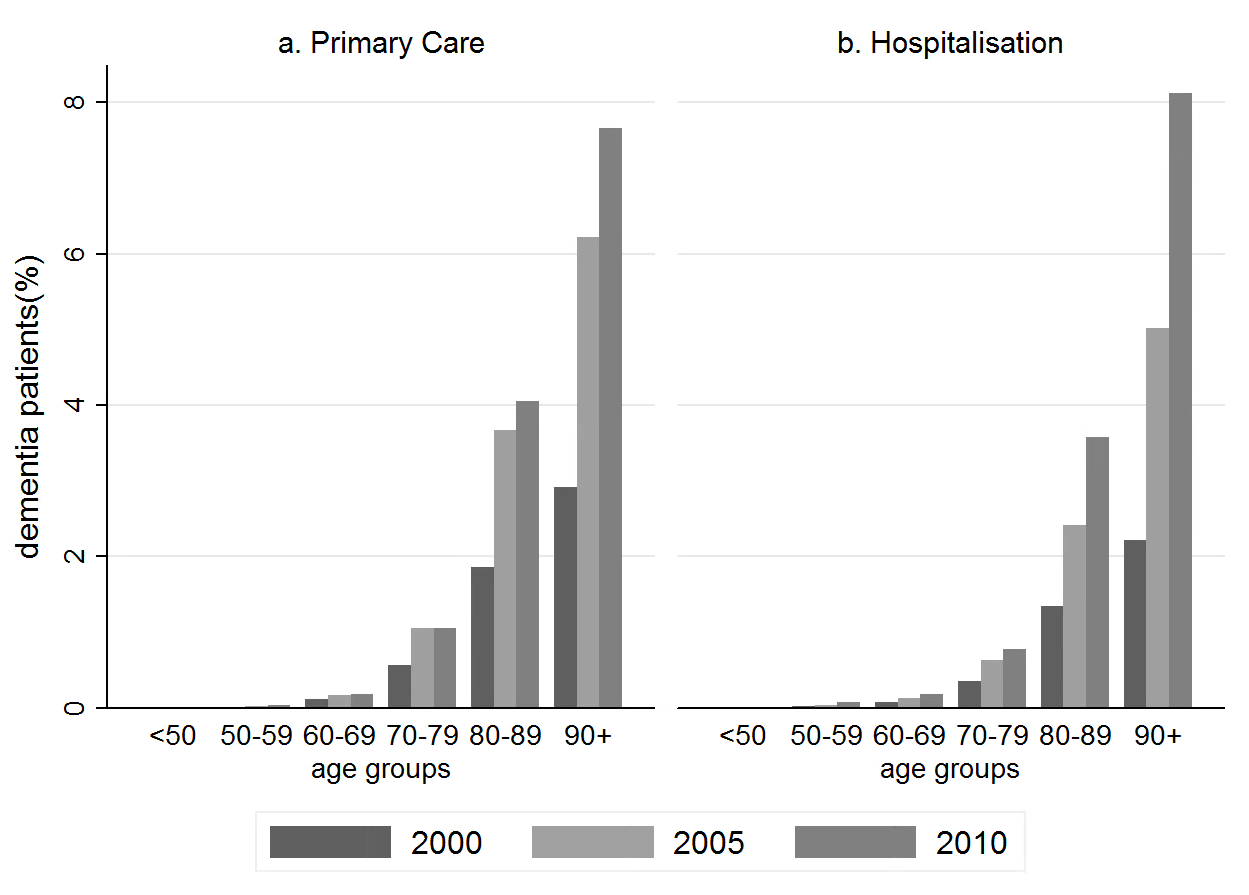
**

Figure note: Point prevalence estimated on 1^st^ July 2000 and 2005 and 1^st^ January 2010
